# Understanding community-based mental health interventions among migrant workers in Singapore

**DOI:** 10.1007/s44192-024-00092-3

**Published:** 2024-09-30

**Authors:** Theophilus Kwek

**Affiliations:** Faculty of Life Sciences and Medicine, MSc Public Health (Global Health), King’s College London, London, UK

## Abstract

**Background:**

Migrants in Singapore face unique mental health risk factors and barriers to formal care. Within this context, the Migrant Writers of Singapore (an arts collective) has organised a community-based intervention to address mental health needs, the Mental Health Awareness and Well-Being Festival.

**Aim:**

To understand migrants’ motivations for organising and participating in the Festival as a form of community-based mental health support, as well as their perspectives on the role and effectiveness of such interventions.

**Methods:**

Thematic analysis of semi-structured interviews conducted in October and November 2023, with 10 members of MWS involved in the Festival.

**Results:**

Interviewees were primarily motivated by personal experiences of giving or receiving peer support, or finding relief through MWS’s arts-based activities; and to a smaller extent by the need for greater mental health awareness among migrants. Interviewees saw the value of community-based interventions in: (i) easing loneliness, (ii) establishing solidarity, (iii) facilitating communication in help-seeking, and (iv) building longer-term social networks.

**Conclusions:**

Findings suggest that community-based interventions may be an enabler of peer support, and help address underlying mental health risk factors. Arts-based activities can enhance these interventions, though further research is required to evaluate concrete outcomes, and ascertain the wider applicability of these findings.

## Background

Mental health risk factors faced by migrant workers in Singapore^1^ have been extensively documented. In addition to debts from agency and recruitment fees, many male migrants – generally employed in the construction, maritime and process sectors – work long hours in dangerous conditions, producing a “heightened context for workplace accidents” [2]. Other sources of distress include salary and injury disputes, as well as the threat of losing their Work Permits, identified as one of the “most important proximate social determinants of predicted mental illness” [3]. Conversely, female migrants, generally employed as domestic workers, often experience feelings of entrapment and stasis due to blurred boundaries between ‘work’ and ‘rest’, and are vulnerable to physical or verbal abuse with limited recourse [4]. Given Singapore’s ageing demographic, many also shoulder disproportionate stress from caregiving for the elderly [5].

These issues were further accentuated by COVID-19 [6, 7]. Male migrants bore the brunt of a large-scale outbreak in Singapore’s migrant dormitories, where the infection rate rose to four hundred times higher than the local community [8]. Prolonged restrictions on movement and work worsened financial anxieties and led to increased symptoms of depression and stress [9]. As for female migrants, movement restrictions – which entailed spending ‘off’ days in employers’ homes – compounded feelings of fear and constant surveillance [10]. To its credit, the Government made significant efforts to alleviate these burdens [11, 12], as well as to expand awareness and support for migrants’ mental health issues more generally [13]. However, many migrants still face long-standing barriers to accessing mental health services [14, 15], including cost, language, accessibility, and information asymmetry [16, 17]. These are exacerbated by stigma around mental health within the migrant community, which prevent many migrants from seeking help or information from family and friends despite burgeoning awareness of the risks [18], and in Singaporean workplaces more generally [19].

With formal support being out of reach for many migrants, it is worth understanding how migrants themselves perceive and seek to address mental health risks. Recent studies have stressed the importance of a “bottom-up perspective” in allowing migrants’ health narratives and practices to shape inclusive public health interventions [20], and ultimately help to counteract the “othering” of migrants in public health systems, as well as public discourse more generally [21]. In this vein, several studies have examined the coping mechanisms of male and female migrants at the individual level [22, 23]. A smaller number of studies have also sought to determine the effectiveness of equipping migrants to deliver peer-to-peer mental health support [24]. However, literature remains scarce on how migrants understand the role of larger-scale community-based interventions to tackle shared mental health challenges, and their personal motivations for contributing to such efforts.

One such intervention is the Mental Health Awareness and Well-Being Festival, organised annually by the Migrant Writers of Singapore (MWS) since 2021 with the stated intent of raising awareness of mental health risks, and reducing stigma around help-seeking within the community [25]. An arts collective comprising male and female migrants from diverse backgrounds, MWS hosts a range of mental health-focused activities before and during each Festival, including arts-based workshops and competitions, dialogues with mental health professionals, and storytelling or ‘human library’ sessions to share their mental health experiences. Held under COVID-19 restrictions, the first edition of the Festival in 2021 saw 50 attendees on-site and was live-streamed on Facebook, garnering around 1,700 views [26]. Subsequent editions of the Festival in 2022 and 2023 were able to accommodate larger crowds, though exact numbers were not tracked by the organisers. Unlike most other Singaporean-led initiatives, the Festival is one of the only sustained, migrant-led interventions to explicitly address mental health needs in the community.

This study takes advantage of the unique lens afforded by the Festival to gain a better understanding of perspectives from within the migrant community on community-based support, which could inform and complement public health efforts to address migrants’ mental health needs. Specifically, this study aims to investigate (i) migrants’ motivations for organising and participating in the Festival; and (ii) how they understand the role and effectiveness of such community-based support (including in relation to other interventions e.g. peer support, formal mental health care).

## Methods and ethical considerations

This study adopted a qualitative design, relying on thematic analysis of semi-structured interviews with MWS members involved with the Festival. Thematic analysis, as described by Braun and Clarke [27], is a methodology designed to minimise the impact of researcher bias, and was chosen to foreground migrants’ perspectives and agency within the findings.

### Sampling

To obtain the richest perspectives on the research questions, interviewees were purposively sampled to prioritise those involved in organising one or more iterations of the Festival, followed by those in more ad hoc roles (e.g. participants or volunteers). This was facilitated by the author’s personal contact through past volunteering with MWS, which also created trust and rapport with the interviewees.^2^ All those identified were approached via WhatsApp (which is typically used for communication among MWS members), and invited to participate on an entirely voluntary, confidential and unpaid basis. To allow for willing and voluntary participation, multiple explicit opportunities were provided for interviewees to opt out, including after the initial invitation, and after hearing more information about the scope and purpose of the study. None of the individuals approached declined to be interviewed.

### Interviews

Altogether, 10 migrants were interviewed between mid-October and end-November 2023, all of whom had been (i) members of MWS for at least a year, and (ii) involved with any of the past iterations of the Festival (i.e. 2021–2023) as organisers or participants. The study excluded Singaporean volunteers, who form a small minority of MWS members, as well as migrants who were unable to converse fluently in English, so as to facilitate data analysis. Two interviews were conducted online with migrants who had returned to their home countries. The remainder were held at Sing Lit Station, a literary non-profit whose premises are regularly used for MWS meetings, and hence regarded as safe and comfortable by the interviewees. An initial set of indicative questions and prompts was developed based on the lines of inquiry above (see ‘Background’), which were updated ahead of each interview to clarify or expand on areas which other interviewees had raised (examples and justifications for such changes can be found in the Box below). This was done without substantively altering the focus or flow of the interviews, so as to allow for closer comparisons between different interviewees’ responses.

**Table:** 

1. Could you describe your involvement in the Mental Health Awareness and Well-Being Festival of XXXX (“year”)? *Further prompts: how the interviewee came to be involved, the nature and extent of involvement, whether this extended over multiple years, and if so, how their involvement changed. (In addition, to prompt responses on how the interviewee felt when learning about the Festival and being asked to take part.)*	
2. Why did you decide to be involved in this way? *Further prompts: the interviewee’s intents and motivations for their involvement in the Festival, including whether these changed over time. (In addition, to prompt responses on how specific aspects of the Festival, including its arts-based activities, may have stood out to the interviewee.)*	
3. Are you aware of other initiatives by migrant workers to promote mental health within the community? *Further prompts: whether the interviewee may have been similarly involved in these other initiatives, and why/why not. (*Note*: This prompt was deprioritised in later interviews, given that none indicated that they knew of similar initiatives.)*	
4. How would you describe the impact of these initiatives on participants? For example, would you describe these initiatives as “effective”, and why? *Further prompts: how interviewees conceive of the “effectiveness” of a mental health intervention, and the relative importance of such interventions vis-à-vis other health-related initiatives in the migrant community.*	
5. With respect to the Festival and any other initiatives you have been involved in, how would you make them more “effective”? *Further prompts: what interviewees would want in an ideal, effective community-based intervention. (To also invite any thoughts or reflections on the overall importance of community-based mental health interventions.)*	

The interviews, which lasted between 50 and 80 min, were conducted in English and audio-recorded with the interviewees’ informed consent. These recordings were then transcribed, and compared with the author’s contemporaneous notes to confirm points of emphasis (e.g. conveyed through interviewees’ body language). Several interviewees were contacted after the initial interviews for minor factual clarifications on points raised in their transcripts (e.g. length of time spent in Singapore). Following this, interview transcripts were carefully examined to establish preliminary themes around the study’s research questions, then re-analysed and coded to identify the most frequently recurring ideas within each theme, as well as to clarify the main findings in greater depth.

Subsequently, both the analysed data and full quotes used were shared with key interviewees to verify the descriptive and interpretive validity of the findings, in accordance with McKim’s [28] proposed approach to member checking. The interviewees agreed with the themes identified and stated that they resonated with the quotes chosen to exemplify each point. None of the interviewees requested for any data or characterisations to be amended or removed.

### Ethical considerations

Migrant workers in Singapore are generally considered a vulnerable population, owing to their social isolation, employment constraints, and other factors [29]. In addition to the measures taken to foreground the interviewees’ agency and voices above, every effort was taken to avoid causing inadvertent harm or distress, as well as to avoid implicating interviewees’ relationships with employers and others. For instance, questions were designed to focus primarily on the Festival, rather than interviewees’ own mental health experiences or work-related grievances, though some interviewees did ultimately cite mental health challenges they had previously faced in explaining why they decided to support the Festival. Interviewees could also request to postpone or stop the interviews at any point if they were experiencing distress, though none eventually chose to do so.

Data collection was handled by the author alone, observing strict confidentiality, and with transcripts retained in a secured device solely for the purpose of analysis. Informed consent was obtained from all interviewees. Finally, all interviewees were identified by pseudonyms in the transcripts and subsequent reporting, and quotations used with interviewees’ permission. Formal ethics approval was obtained for this study from King’s College London’s Research Ethics Office (reference no. LRU/DP-23/24–39,775), and the study was conducted in accordance with all relevant guidelines and regulations.

## Findings

### Participant characteristics

While the sample was not intended to be representative of the migrant population, interviewees’ backgrounds (Table 1) were typical of migrants in Singapore, with a skew towards Filipina domestic workers, reflective of the broader membership of MWS. Majority had spent between 11 and 20 years in Singapore.

**Table 1:** Key characteristics of interview sample

Sex	
Male	3
Female	7
Countries of origin	
Indonesia	2
Bangladesh	3
Philippines	5
No. of years in Singapore	
≤ 10 years	2
11–20 years	6
≥ 21 years	2
Total	10

In view of the abovementioned sensitivities, individualised details are not provided to avoid identification of the interviewees and/or the unmasking of pseudonyms used

### Motivations for involvement in the festival

Migrants involved in the Festival were predominantly driven by personal experiences: either of giving or receiving peer support in the community context, or of obtaining relief for their mental health through MWS-organised activities.

**1. Experiences of giving or receiving peer support:** When asked why they decided to be involved, a majority (n = 6) of interviewees shared examples of fellow migrants whom they had met through MWS activities, and subsequently supported through distressing situations, especially under the pressures created by COVID-19 (e.g. disputes with employers, acute loneliness, or financial troubles leading to anxiety and suicidal ideation). They acknowledged the challenge and responsibility of *rendering* such peer support, and saw the Festival – especially the arts workshops and dialogues with mental health professionals – as a way of equipping themselves and others to do so more effectively. For instance, while ‘Sara’ had previously explored skills-based courses (e.g. in financial literacy and computer skills) offered by other nonprofits, she eventually joined MWS and began supporting its activities because they equipped her to “motivate and help my friends”, especially those facing difficulties with their employers. As she put it:

*“It is helpful for me, and also helpful for [my friend], because I learn a lot from what MWS doing. How to manage our own self when we run into difficulty, this is [something] we can give to others as well.”*

As for ‘Coralyn’ (not among the 6), it was *receiving* peer support through MWS after the demise of her father in 2021, that prompted her to volunteer. In her words:

*“I open up to my friend, she is my mentor and she is also a volunteer here in MWS. She encourage me to join them, and share my experiences and [try] different activities which help me to cope with everything [...] It’s really affected me so much that I need to come out of my comfort zone and share this [with] other people, to help them manage also.”*

Her experience of effective peer support and desire to extend this to others thus formed a powerful motivation for her own participation in the Festival.

**E2. Experiences of finding relief through similar activities: **Another motivation cited by half the interviewees (n = 5), was having found relief from their own distress through MWS activities, and wanting to create a similar outlet for others. They highlighted the arts and poetry workshops, a mainstay of the Festival, as allowing them to articulate the emotionally draining experiences they had been through, and gain self-confidence in the process. Indeed, some, like ‘Janet’, were able to pinpoint specific arts activities that convinced them to lend the Festival their support:

*“I joined a photography and literary walk around October 2019, [and] it really gave me a realisation about the importance of taking care of our mental health, the arts and literature really helps. That’s when I realised I want to be part of this community.”*

This resonated with how another organiser, ‘Hassan’, saw this aspect of the Festival:

*“Everyone has a story, but they don’t have a space to tell their story. We found that being able to tell their story is a very big part to recover the mental sickness. So we include storytelling, poetry, in our Festival.”*

Some interviewees affirmed this by sharing their observations of how the Festival’s arts-based components had also allowed other attendees to process and express their emotions. An important part of this was being able to perform or share their negative experiences through art, and receiving affirmation from an audience. As one attendee-turned-organiser (‘Jessie’) shared, the poetry workshop and open mic had given one of her friends a cathartic opportunity “to say ‘I’m done’, and move to the next chapter of their lives” – an experience she hoped to extend to other domestic workers.

Besides these personal experiences, a smaller number of interviewees (n = 3) cited the need to improve mental health awareness among migrants generally as their motivation for supporting the Festival. All three pointed to reports of completed or attempted suicides by migrants as evidence of the scale of this need, corroborating this from their own observations. According to ‘Mohan’, who lived in a dormitory with around five thousand men:

*“Every night, I hear a lot of people discussing their problems, in Bengali, in Hindi, in English, in Malay, in Tamil, so many issues that they can’t share with everyone. All these things were inside my mind, when I heard the call [to volunteer]. Some guys are aware about mental health, but […] if I’m not wrong, there was another suicide case last month at the dorms. That still points us that we can do a lot of things for them.”*

As one of the earliest MWS members involved in the Festival, ‘Mohan’ saw it as his personal mission to broaden its outreach in the process sector, and then to “all the dorms” in Singapore. Nearly all the interviewees echoed this sentiment when asked about the overall “effectiveness” of the Festival, equating this with raising awareness of mental health among as many migrants as possible.

### Importance of community-based support

Beyond the Festival, interviewees also expanded more generally on the value of community-based interventions in mental well-being, particularly in comparison with professional mental health care, with four themes emerging:

**1. Easing loneliness:** Interviewees alluded to “being alone” as the fundamental condition of being a migrant in Singapore, or as the primary factor exacerbating feelings of anxiety or distress. ‘Nellie’, for instance, identified “loneliness” and “longing for my family” as the two main features of her time in Singapore, spanning more than a decade. For these interviewees, community-based interventions were crucial in demonstrating, in ‘Jessie’s’ words, that:

*“You are not alone in this foreign land. [There are others] who know the feeling of being away from our loved ones, and know the feeling of being migrants. We have the same situation […] It’s very helpful to have this kind of support.”*

Independent of whether they received professional help, alleviating this baseline loneliness helped migrants put their own problems in perspective, and draw comfort from those who had similar experiences.

**2. Establishing solidarity:** Related to the issue of loneliness was that of self-doubt, frequently compounded by stigma or a lack of awareness about mental health. As ‘Belle’ (who was also a helpline volunteer with another non-profit) explained, migrants who experienced mental health symptoms would decline professional help, asking, “I’m not crazy, why should I go to a psychiatrist”? In this context, community-based support from fellow MWS members could lend reassurance, and avoid a further loss of confidence and self-esteem. In ‘Belle’s’ words:

*“We can tell her that we are all domestic workers, we’ve been there, we’ve experienced that […] That’s the difference in getting professional help, and hearing it from a friend. Asking a professional will make them question ‘what’s happening to me’, so sometimes it can make the matter worse”.*

**3. Facilitating communication:** In addition to self-doubt, interviewees highlighted other non-cost barriers preventing migrants from seeking professional help for mental health issues, including the underlying gulf in communication (arising from differences in language, status, etc.) between migrants and locals. As ‘Mohan’ said:

*“Even if there is no fees involved, the worker will still be confused to talk, because they are not really familiar with Singaporean doctors […] the social bonding is still not there”.*

Likewise, ‘Ellie’ shared that migrants often felt that activities or support systems organised by locals were “not for [them]”, but were more “confident and comfortable” sharing their problems in a “migrant to migrant” setting. It was thus important for migrants to be able to access support from someone who could genuinely claim to have “come from their level”, rather than a local professional who would be “totally unknown” (‘Mohan’).

**4. Forming longer-term networks: **Finally, interviewees credited the sustained and recurrent nature of MWS activities for establishing social networks that served as a source of stress relief, and gave them a sense of personal and shared significance. For instance, ‘Sara’ shared that:

*“Before I joined MWS, I felt like I’m nobody: nobody knew me, I had not many friends. [By] joining the activities, I can forget about the problems I am going through […] and we can help each other grow and understand more about[our] own selves”.*

MWS’s wider network also allowed interviewees to access more tangible support. As ‘Jessie’ shared, those who approached MWS with more complex work-related issues could be “referred to other NGOs who can help them professionally”, allowing them to have their needs met more holistically. Again, independent of professional care, this aspect of community-based support was crucial for maintaining mental well-being in the longer term.

Regardless of these positive views towards community-based support, several interviewees stressed that it was still important to tackle barriers to professional care for migrants: especially where mental health challenges were severe, or where fellow migrants were not equipped to render effective peer support. Even so, they saw the Festival as a helpful platform to increase their exposure to mental health professionals and resources, allowing them to direct their peers towards more formal care.

## Discussion

Existing studies on peer support for mental health among migrant workers have generally focused on the effectiveness of peer-to-peer mutual aid, or para-professional training in peer support skills [30]. Few have examined the role of community-based support and collective interventions, especially where initiated by the migrant community rather than public health or non-governmental agencies; or the relationship between peer-to-peer support and larger-scale initiatives like the Festival. This study thus sheds new light on a number of areas.

First, the findings indicate that the opportunity to provide or receive peer support is a key component of why migrants choose to participate in community-based mental health interventions, as well as the value they see in these initiatives. Personal experiences of receiving or giving peer support were prominent motivations for some involved in the Festival; and in describing the value of community-based support more generally, two of the four themes that emerged (‘establishing solidarity’ and ‘facilitating communication’) were also directly related to how effectively community members could render peer-to-peer support, as compared to a professional. However, community-based interventions should not simply be seen as peer support on an expanded scale, and it is worth noting how the Festival figured in participants’ accounts specifically as providing the *social context* within which they could encounter fellow migrants and thus extend support to them, or the *skills and know-how* to render peer support more effectively. From another perspective, the Festival can be seen as creating a valuable space within which the giving and receiving of mental health peer support is normalised, with less of the stigma that may be felt in other settings. With the growing momentum behind mental health peer support across various settings in Singapore [31], future efforts to promote peer support among migrants could consider leveraging existing community-based interventions as a catalyst, or adopting aspects of these interventions to create a conducive environment for peer support, such as inviting community members to co-design sessions premised on mutual recognition and reassurance.

Notwithstanding the above, the findings also indicate that community-based initiatives have significant value beyond being a conduit or enabler for peer support. Interviewees highlighted ‘easing loneliness’ and ‘building longer-term social networks’ as two equally valuable aspects of community-based interventions, both of which go beyond facilitating support for immediate mental health challenges, to addressing two fundamental conditions experienced by almost all migrants – that of being separated from pre-existing support networks in their home countries, and being isolated or unable to form meaningful connections at their places of work and rest. These views resonate with other studies which have identified social isolation as a major stressor for migrants, and establishing social networks as a key coping mechanism [32, 33]. Separately, interviewees also saw the Festival as an important platform for raising awareness generally about mental health among migrants, seen as a first step to self-care and help-seeking, and helping to avoid more severe distress or self-harm, which has been associated with the barriers to care that migrants more generally face [34]. Taken together, these findings suggest that even in the absence of effective peer support, community-based interventions have an important role in addressing migrants’ underlying needs, and public health agencies can consider how to encourage or resource them in their own right. Comparative studies can also be undertaken to determine if the presence and persistence of such community-based interventions can be associated with lower levels of stigma around mental health among similar underserved communities in different contexts.

Finally, the findings indicate that arts-based activities, especially those which facilitate emotional release and the sharing of personal experiences, can contribute to the success of community-based interventions among migrants. Studies have shown that community-based interventions including art and music therapy alongside other elements (e.g. mindfulness) can effectively address anxiety and depression among other target groups in Singapore, like the elderly [35]. However, no equivalent research has been conducted among migrants. While not the core focus on this present study, its findings nonetheless suggest that arts-based activities are viewed by a diverse group of migrants as helpful in facilitating emotional relief through self-expression and catharsis; and that these activities have been sufficiently positive and galvanising for those involved to organise them regularly, and encourage others to participate. Future studies can focus on whether specific arts-based activities may lend themselves better to supporting migrants with specific needs, and also evaluate the therapeutic effectiveness of such arts-based activities, in order to arrive at a firm recommendation on whether to incorporate them in mental health interventions for migrants more generally, especially given the resources and expertise required to organise them at scale.

### Limitations

This study’s sample size and method were designed to support its research questions, which were centred around one specific intervention (the Festival). Given its focus on an intervention organised by an arts collective, the study also necessarily drew from a self-selecting group (i.e. established members of MWS with pre-existing interest in the arts). As such, the applicability of its findings to a wider population of migrants, including those who have spent less time in Singapore, as well as those from other socio-linguistic backgrounds (e.g. Chinese workers, who are generally less fluent in English and hence under-represented among MWS’s membership) [36], will have to be separately ascertained. Though steps were taken (e.g. member checking) to minimise researcher bias, the fact that this study was conducted by a single author may have skewed the interpretation of some findings. Moreover, the author’s identity as a past MWS volunteer, though helpful for recruitment, may have introduced further bias (e.g. support for MWS’s aims and methods) to the data analysis. In view of these shortcomings, further study will similarly be required to validate its findings against a wider set of perspectives towards community-based mental health interventions among migrants in Singapore.

Nevertheless, this study has opened new lines of inquiry around migrant-led, community-based interventions which have hitherto not been investigated in Singapore. In due course, it is hoped that the above recommendations for policy action and further study can build towards a fuller recognition of the role of such interventions in addressing mental health challenges among this vulnerable population.

## Conclusion

A holistic understanding of the mental health situation among migrants in Singapore should not only account for individual coping mechanisms and peer support, but the role of community-based interventions in addressing shared mental health needs. This study represents an initial investigation into the motivations behind one specific community-based intervention, as well as migrants’ perspectives on the value of such interventions more generally. It finds that community-based interventions can be an enabler of peer support, and also help address underlying mental health risk factors such as loneliness, suggesting that future public health efforts can place more focus on the role of such interventions. Separately, it also finds that arts-based activities can contribute to positive perceptions of community-based interventions among migrants, although further research is applicability of these findings.

### What is known?

Migrants in Singapore face mental health risk factors arising from their professional and social conditions, exacerbated by COVID-19 and long-standing barriers to care. Other studies have examined the role of individual coping mechanisms and peer-to-peer support, but not community-based interventions, in addressing these needs.

### What is new?

The study uncovers why migrants choose to participate in community-based mental health interventions, including how peer support and arts-based activities contribute to the perceived value of such interventions. 

### What is the impact?

Findings suggest the need to recognise and build on community-based interventions in providing effective mental healthcare for migrants.

## Data Availability

Data is provided within the manuscript.

## Footnotes

1 This paper focuses on migrant workers on Work Permits, numbering around 111 million as at December 2023. These include, but are not limited to, around 286,300 female domestic workers, as well as 441,100 male workers in the construction, maritime and process sectors [1].

2 The author is a male, English-speaking Singapore citizen with no formal relationship to any of the interviewees, apart from prior familiarity as a fellow volunteer at MWS activities.
